# Comparison of the effects of reef and anthropogenic soundscapes on oyster larvae settlement

**DOI:** 10.1038/s41598-024-63322-2

**Published:** 2024-05-31

**Authors:** Sarah Schmidlin, Clea Parcerisas, Jeroen Hubert, Maryann S. Watson, Jan Mees, Dick Botteldooren, Paul Devos, Elisabeth Debusschere, Pascal I. Hablützel

**Affiliations:** 1 Flanders Marine Institute (VLIZ), Jacobsenstraat 1, 8400 Ostend, Belgium; 2 Department of Biology, Ghent University, Krijgslaan 281, Campus Sterre S8, 9000 Ghent, Belgium; 3 Department of Information Technology, Ghent University, Technologiepark-Zwijnaarde 126, 9052 Ghent, Belgium; 4 Institute of Biology, Leiden University, Sylviusweg 72, 2333 BE Leiden, The Netherlands; 5 Marine Animal Ecology Group, Wageningen University, Wageningen, The Netherlands; 6 Groningen Institute for Evolutionary Life Sciences, University of Groningen, Groningen, The Netherlands; 7 Coastal Systems, Royal Netherlands Institute for Sea Research (NIOZ), Den Hoorn, Noord Holland The Netherlands; 8 Biology Department, Vrije Universiteit Brussel, Pleinlaan 2, 1050 Brussels, Belgium

**Keywords:** Larvae settlement, Underwater noise, Noise pollution, Soundscapes, Settlement cue, Oyster reef ecology, Behavioural ecology, Restoration ecology, Acoustics, Marine biology

## Abstract

Settlement is a critical period in the life cycle of marine invertebrates with a planktonic larval stage. For reef-building invertebrates such as oysters and corals, settlement rates are predictive for long-term reef survival. Increasing evidence suggests that marine invertebrates use information from ocean soundscapes to inform settlement decisions. Sessile marine invertebrates with a planktonic stage are particularly reliant on environmental cues to direct them to ideal habitats. As gregarious settlers, oysters prefer to settle amongst members of the same species. It has been hypothesized that oyster larvae from species *Crassostrea virginica* and *Ostrea angasi* use distinct conspecific oyster reef sounds to navigate to ideal habitats. In controlled laboratory experiments we exposed Pacific Oyster *Magallana gigas* larvae to anthropogenic sounds from conspecific oyster reefs, vessels, combined reef-vessel sounds as well as off-reef and no speaker controls. Our findings show that sounds recorded at conspecific reefs induced higher percentages of settlement by about 1.44 and 1.64 times compared to off-reef and no speaker controls, respectively. In contrast, the settlement increase compared to the no speaker control was non-significant for vessel sounds (1.21 fold), combined reef-vessel sounds (1.30 fold), and off-reef sounds (1.18 fold). This study serves as a foundational stepping stone for exploring larval sound feature preferences within this species.

## Introduction

Identifying a suitable habitat prior to permanently transitioning to a benthic life stage is critical for future survival, growth, and reproduction in many marine invertebrates with planktonic larvae. These species therefore utilize of a variety of environmental cues, enabling them to identify promising settlement locations^1^. Experimental research has shown that in some species, a single cue can induce settlement and subsequent metamorphosis^1–3^. But in many other species larvae may respond to more than one cue, *Crassostrea virginica* larvae for example respond similarly to chemicals released by conspecific adults, and chemicals released from mature biofilms^4^. Cues can have chemical and physical origins, and while some types of cues require contact with a prospective settlement location such as cues associated with shells of conspecifics adults or cues from topographical features of a substrate^5,6^, other cues may act over larger distances to guide larvae to their preferred habitat such as those released by waterborne conspecific chemicals^1,7^. More recently, acoustic cues have been identified as drivers of larval settlement^8,9^. As sound propagates relatively fast and far underwater, it serves as an efficient signal transmission medium. For many marine species, sounds can convey specific events, such as presences of a predator or a mating opportunity^10,11^. But, collectively, soundscapes can also convey overall quality and suitability of an environment for a species^12,13^. Research on acoustic cues informing larvae about optimal habitats has only been established relatively recently^8,9^. In certain invertebrate species with a settlement/metamorphosis stage, including crabs, corals, and bivalves, acoustic cues have been shown to affect larvae swimming direction^8,9^, settlement rates^9,14^, and amount of time a larvae takes from entering competency to completing metamorphosis^15–17^. In general, it seems that natural environmental sounds can convey information to invertebrate species in that environment^18^. In coral and bivalve reefs, larvae seem to be attracted to soundscapes from healthier reefs, which produce louder and more acoustically complex sounds compared to less healthy reefs which are much quieter^14,19^. However, the particular characteristics of reef soundscapes (e.g. sound pressure level (SPL), specific frequencies, complex mixtures of these or other acoustic characteristics) that elicit settlement behaviors remain unclear.

Anthropogenic sounds may interfere with or mask natural marine soundscapes^12^. Vessel noise can mask important sound cues resulting in poorer orientation toward reef sounds for some species of fish^20,21^, and cause coral larvae (planulae) to delay settlement^22^. Anthropogenic noise can not only disrupt or reduce larval settlement but may also be (mis)interpreted as a cue to settle in some taxa^16,17,23–25^. Vessel noises have been shown to increase some larvae settlement, including in mussel *Perna canaliculus*^16^ and *Mytilus edulis*^24^. Why anthropogenic noises are interpreted as settlement cues in some taxa but are repulsive to others is unknown. The reaction to anthropogenic sounds may depend on the acoustic profile of a species' preferred habitat and which features of this profile are responsible for attraction^17,23,26,27^.

The oviparous true oyster *Magallana gigas* is an important reef-building ecosystem engineer^28^ and a valuable species for aquaculture^29^. But in many areas it is invasive and considered a biofouling pest that poses a threat to local species and ecosystems^30,31^. There is considerable interest in settlement preferences of this species for both bolstering as well as reducing recruitment^32^. In recent years, there has been a global effort to restore oyster reefs, as widespread habitat destruction have left native historical populations decimated^32^. Availability of settlement cues is crucial for reef sustainment, with some reports suggesting that these cues may outweigh other recruitment factors such as local hydrodynamics, and larvae supply^33,34^. A recent revelation that oyster *Ostrea angasi* not only settle more rapidly but also exhibit horizontal swimming movements toward sound sources underscores the significance of soundscapes as a navigation tool for larvae^9^. So far, larvae of *M. gigas* have not been studied for their response to acoustic settlement cues (but see Stocks et al.^23^) for an account of swimming activity in response to natural and vessel sounds). *M. gigas* adults have been studied for their sense of hearing, and were found to react by valve closure to pure tones in the range of 10 to 1000 Hz at minimum energy of 122 dBrms re 1 μPa^35^. While these adults are studied for their pure tone reactions, the range of hearing of these larvae have not yet been identified. Other true oysters with relevant experimental data are the closely related and also oviparous *C. virginica,* and the more distantly related larviparous *O. angasi*. Experimental studies have shown that both *C. virginica* and *O. angasi* larvae prefer louder reef sounds in frequency ranges 1.5–20 kHz over quieter off-reef playbacks or no-sound controls^7,9,36^.

In this study, we present the results of laboratory-playback based settlement experiments on the role of acoustic cues in settlement and metamorphosis of *Magallana gigas*. Firstly, we were interested in the importance of oyster reef sound compared to off-reef sound. Secondly, we wanted to know whether vessel noise attracts or repels pediveliger larvae. To do so, we exposed larvae to different vessel and reef sounds as well as off-reef and no-speaker controls. Finally, we subjected the larvae to vessel and reef sounds simultaneously to find out whether vessel noise modifies, or masks oyster reef sound cues.

## Methods

We conducted laboratory tank-based playback experiments to investigate whether oyster species *Magallana gigas* larvae alter their settlement response in reaction to sounds emitted by conspecific oyster reefs and vessels. Sound recordings were obtained from two regions within the North Sea, and acoustic spectral features were analyzed based on recordings made within experimental tanks.

### North Sea soundscape measurements

**Figure 1:**
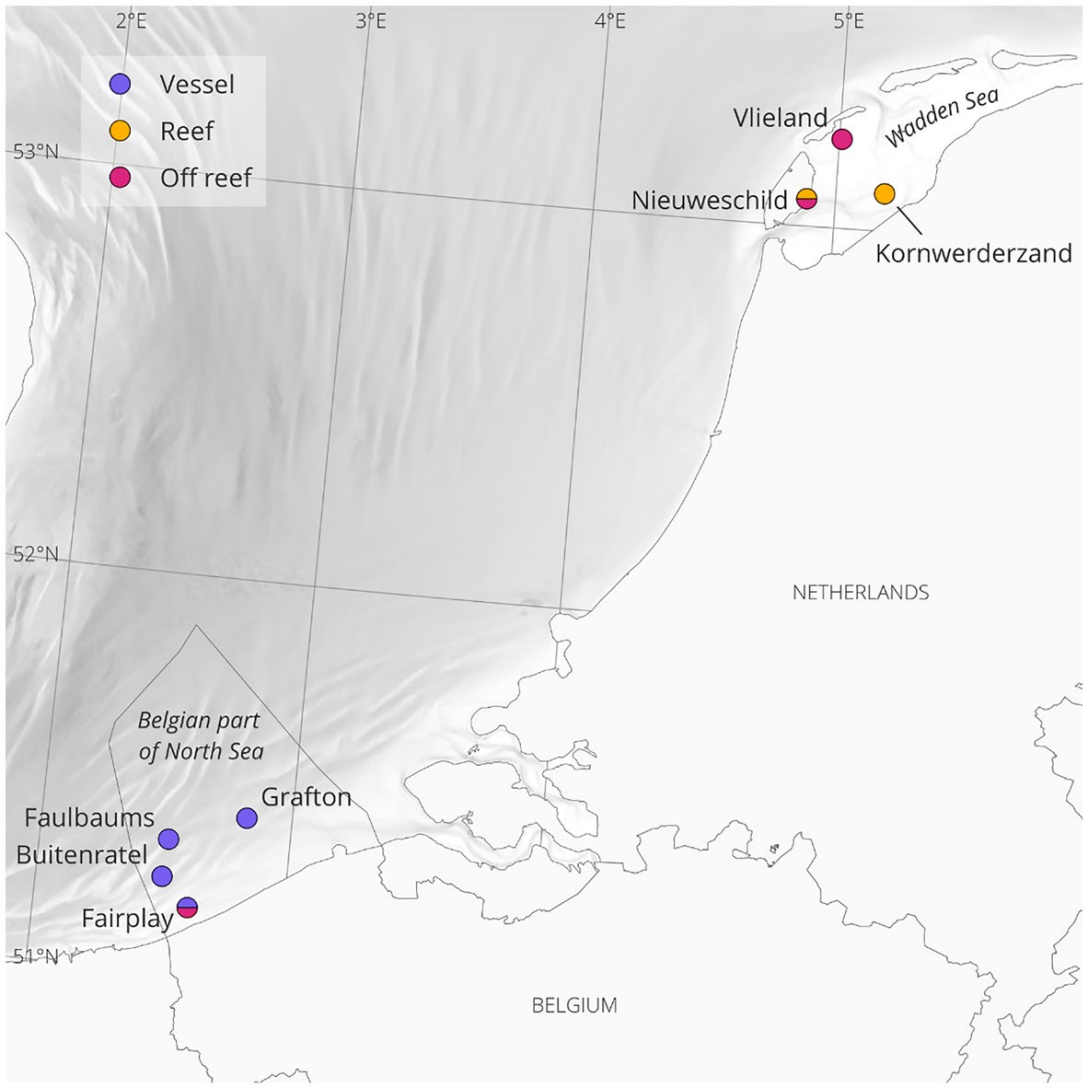
Distribution of the locations where underwater sound data were collected. Colors represent which treatment was collected there. Sounds acquired in locations with two colors were used for different treatments. Map made by maps@vliz using QGIS version 3.30.0-'s-Hertogenbosch (https://qgis.org/en/site/).

All recordings used during the experiment were recorded in two regions of the North Sea: the Southern Bight near the Belgian coast and in the Dutch Wadden Sea (see Fig. 1).

In the Dutch Wadden Sea, recordings at subtidal reef sites were collected with hydrophones (SoundTrap 300STD; Ocean Instruments, NZ; sampling rate 24 kHz; manufacturer-calibrated; set at low gain, see Table S1 for details). Hydrophones were suspended in PVC frames anchored to the seafloor and a subsurface buoy ensured that the hydrophone was positioned approximately 1 m above the seafloor in water depths ranging from 2 to 5 m. These were deployed from a small boat, and left to record continuously for two weeks or the maximum battery life. Reef sound recordings used for this experiment were taken at two subtidal oyster reefs in the Marsdiep tidal basin of the eastern Wadden Sea (Table S2). An off-reef sand recording used in this experiment was obtained from a control recording of an artificial reef monitoring in the Eierlandse Gat tidal basin, near the island Vlieland.

For the Belgian part of the North Sea, underwater sound data used were recorded as part of the LifeWatch Broadband Acoustic Network^37^. These data are collected continuously in different fixed locations in the Southern Bight. All locations were close to a shipwreck (< 50 m), but there are no oyster reefs present. These data were collected using a RESEA 320 recorder (RTSys, France) together with a hydrophone. For the stations Buitenratel and Grafton, the hydrophone used was a Colmar GP1516M-LP (Colmar, Italy, sensitivity: −168 dB/V re 1 μPa, frequency range −3 dB: 10 Hz to 70 kHz). For the stations Fairplay and Faulbaums, it was a Colmar GP1190M-LP hydrophone (Colmar, Italy, sensitivity: −180 dB/V re 1 μPa, frequency range −3 dB: 10 Hz to 170 kHz). The hydrophone and the acoustic recorder were attached to a stainless-steel structure frame, at 1 m above the sea bed which stood stable on the seafloor. All stations were between 10 and 30 m deep. Deployments lasted between 4 and 6 months.

### Sound treatments and playbacks

Suitable recording files for each treatment were manually selected. Only Belgian recordings from spring and summer were considered, in order to correspond to the recording period of the sounds from the Wadden Sea (reef and off-reef). Reef sounds were only selected when they contained no apparent outside influences (e.g. vessel sounds). For vessel sounds, a fair variability of sounds was selected, from short sounds of distant vessels to longer continuous sounds from vessels operating close by, with no other audible background sounds. All vessel sounds were recorded from locations in the Southern Bight (see Table S2). For reef treatment, sounds used were recorded from the same location in Texel, NL but sound files used during each day of the experiment were selected from different recording dates (see Table S2). For off-reef sounds, two sound files were used recorded from Texel, NL and two sound files were used recorded from non-reef areas in the Southern Bight off the coast of Belgium (see Table S2). Treatments where vessel sounds and reef sounds were played together were created artificially overlaying reef sound files and vessel sound files.

Selected segments were then combined to create one 1 h file per treatment and day. In some cases, the selection led to files shorter than 1 h, so the segments were repeated and combined by applying crossfading with Audacity^38^ to create a 1 h file. When enough recordings were available for 1 h or more, segments were not repeated. To deal with differences in sampling rate and minimum recording frequency between the selected files, all files were filtered using a Butterworth bandpass filter (N = 4) between 20 Hz and 12 kHz. After filtering, all files were downsampled or upsampled to 48 ksps to match the playback requirements. Details of the selected data are listed in Table S2. In total, 3 recordings of reefs from 2 different locations, 4 vessel recordings with several boats on each recording from 4 different locations, and 4 off-reef recordings from 3 different locations were used to represent our treatments.

Throughout the experiment, each treatment group containing sound playback (“reef” “vessel” “reef + vessel” “off reef”) consisted of a separate recording representing the intended environment. The use of multiple sound files of the same treatment was used to strengthen confidence that the sounds were representative of treatments as a whole, and not of a single event. Employing a series of recordings from various sound sources representing the same treatment enhances the extrapolative capacity of a study^39–41^.

**Figure 2:**
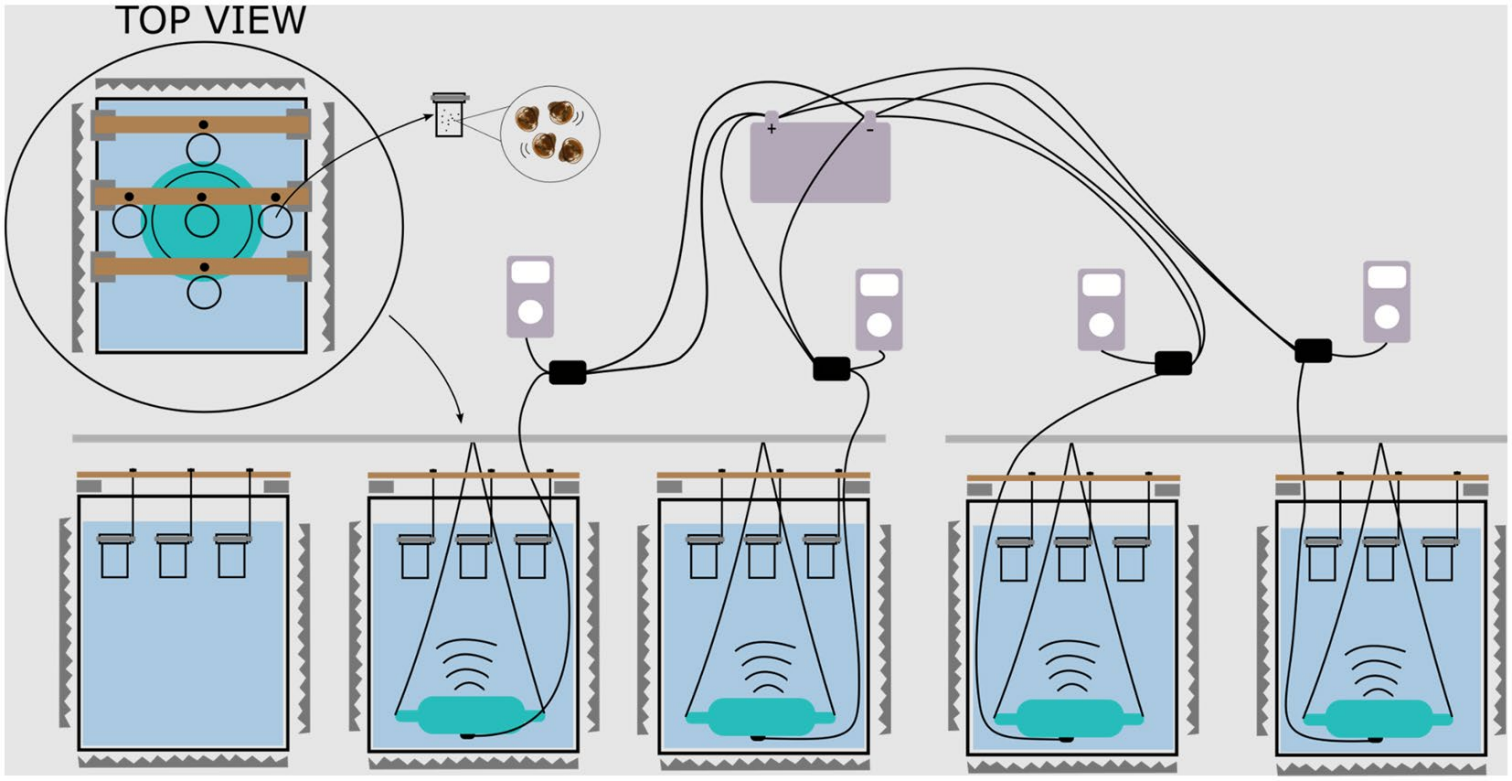
Schematic depicting five tanks. Four of these tanks are equipped with speakers, each of which is connected to a playback device. All speakers and playback devices are linked to a DC battery as their power source. Five 100 ml jars were securely positioned to hang at the same height above speakers. The speakers were suspended within the tanks in a manner ensuring they did not come into contact with the tank's bottom.

The playback set-up consisted of five 100L tanks (49 × 65 × 50.5 cm), separated 20 cm from each other on a rack. Each tank sat upon a 4 cm layer of polystyrene to isolate it from the rack and an additional layer of acoustically absorbent foam (25 mm thick) between the polystyrene and the tank bottom. Acoustic foam was also placed at the tank sides. Four Lubell UW30 Underwater Speakers with custom-made amplifiers, battery-powered to avoid 50 Hz noise or electrical interference from the power grid, were used. Each speaker was connected to one TASCAM playback device which played playback files on repeat. No speaker was placed in the no-sound control (see Fig. 2). This no speaker treatment was added as a second control (aside from the off-reef control) to establish if there were differences between treatments with sound and normal lab conditions. The speakers were hung in the middle of the tank with ropes so they would not touch the tank walls. Larvae were placed inside 100 ml polystyrene jars and these containers were fixed in the same position in the tank for every day of the experiment. These positions were 12.75‬ cm far from the closest jar (distance between outer jars and center jar), and 33.5 from the speakers (see Fig. 2).

For each treatment file, a playback volume was chosen so exposure power spectral density (PSD) would match the sound levels recorded in field as closely as possible. Sound levels specified in literature as typical of reefs (at 1 m from the seafloor) and off-reefs (at 2 km from the reef)^9,42^ roughly matched the chosen levels.

This was done separately for each sound exposure treatment by adjusting the volume in an iterative fashion until the recorded sound levels in tanks were similar to the desired ones. For this purpose, each tank setup was recorded using a TASCAM recorder device together with an Aquarian Scientific hydrophone (AS-1). The hydrophone was placed inside a jar without touching the walls, simulating the position where larvae would be during the experiments. The hydrophone cable was taped to the structure supporting the jars. Recorded sound was converted to sound pressure by using the available calibration data for the hydrophone and the TASCAM recorder. This calibration was cross-checked by comparing it to measurements of the hydrophone used in Hubert et al.^43^.

To measure sound level and acoustic characteristics of each playback received by larvae during the experiment, each treatment was recorded using the chosen playback volume for 1 h (tank recordings) at 48 ksps. This same procedure was used when selecting the playback volume. When recording these 1 h files, all four different sound treatments of that batch were turned on to record possible acoustic crosstalk from the other treatments. Furthermore, the room noise was also recorded using the same protocol when no speaker was active.

For each treatment, several acoustic features were computed for both 1 h recorded tank files and 1 h compiled field files. Acoustic Complexity Index (ACI), Acoustic Evenness Index (AEI), and Acoustic Diversity Index (ADI) were computed using the maad python package^44^, and Power Spectrum Density (PSD) was computed using the scipy python package^45^. The average PSD was computed for three different bands by averaging the spectrum density of all the frequency bins included in the specified frequency band. The parameters used to compute each of the features are summarized in Table S3.

Both ACI and ADI are proxies to quantify acoustic complexity (the higher the number, the more complex), while low values of AEI represent an even sound and higher values represent more uneven sounds. This is not correlated with the ecological concept of evenness, as acoustic evenness refers to an even distribution of sound energy in different frequency bands, and this can be achieved due to a high biodiversity vocalizing at the same time covering all the frequency bands or by constant broadband sounds such as some anthropogenic sounds^46,47^.

### Broodstock and larvae culture

Ten mature adult oysters (five females and five males) were purchased from the Guernsey Sea Farms Ltd (Guernsey, UK) and used to produce larvae. Eggs were fertilized by gonad stripping following FAO guidelines^48^. Fertilized eggs were kept undisturbed in flat bottom tanks for 48 h at 22 °C at a density of ten eggs per ml of filtered seawater (FSW). All seawater used in this experiment was filtered at 0.1 µm and passed through UV light. After 48 h larvae were sieved over 70 µl nylon mesh sieve, rinsed, and transferred to rearing tanks with FSW. Tanks were aerated and kept at 22 °C for the entire duration of larvae rearing. Every two days larvae were sieved over mesh corresponding to the average size of the larvae and the water in the tanks was changed. Larvae were fed a mixture of fresh microalgae mixture consisting of *Chaetoceros muelleri*, and *Isochrysis galbana* (clone T-ISO). For the first 4 days larvae were fed at 40,000 cells/ml water using only *I. galbana* (clone T-ISO). Days 5–12 larvae were fed *C. muelleri*, and *I. galbana* (clone T-ISO) at 100,000 cells/ml at a volume ratio of 1:1. Days 13 + larvae were fed *C. muelleri*, and *I. galbana* (clone T-ISO) at 100,000 cells/ml at a volume ratio of 3:1. Larvae entered their pediveliger stage and became competent to settle at 29 days and were used in settlement experiments starting on this day. Larvae were determined for competence when they had a prominently displayed eyespot and larval foot and were sized at 320–350 μm in diameter.

### Settlement experiment design

The experiment aimed at assessing the effect of sound treatment on larvae settlement. In the experimental design, we were constrained by having only four underwater speakers at our disposition. We therefore repeated the experiment four times over five consecutive days. In each of the four trials, different treatments were assigned to a unique combination of speaker and tank to account for possible speaker or tank effects. The sound treatment was applied at the tank level, making tank the experimental unit. As the experiment has a binary outcome (settled vs. not settled), many sample units (larvae) are needed to accurately assess the treatment effect. Therefore, ten larvae were placed together in jars and five such jars were placed in each tank (see Fig. 2). Larvae were not re-used and for each trial, new pediveliger stage larvae were taken randomly for the same stock. As a consequence, larvae were gradually older through the five day experiment.

### Settlement assays

Experiments took place over five consecutive days (03/03/23–07/03/23) using larvae from the same batch. To control for larvae size, on each experiment day some larvae from culture tanks were filtered between 260 and 300 μm nylon mesh sieves, only larvae retained on the 260 μm sieve were used in the experiment. 10 larvae were gently pipetted randomly into each of the five 100 ml containers per tank and filled with filtered seawater (FSW) and 0.2 g of oyster shells which could act as a settlement substrate. To get a consistent shell topography, shells were crushed using a hammer and crushed shells were sieved between 1.0 and 0.5 mm metal sieve. For each treatment tank, 5 individual containers were used. As all treatments were repeated over 4 consecutive days, 20 jars were used per treatment in total. All trials were conducted in a dark environment at 20 (± 1) °C in a climate-controlled room.

To avoid any air in jars containing larvae, larvae were placed in the jars and the lid was fixed while the jar was fully submerged in FSW. This step was necessary to prevent any distortion of the sounds due to reflection from air bubbles. All FSW used in the experiment had added microalgae *Chaetoceros muelleri*, and *Isochrysis galbana* (clone T-ISO) at 100,000 cells/ml at a volume ratio of 3:1. In a previous study, *M. gigas* larvae increased swimming when exposed to reef sounds, but only if larvae were fed^23^, thus microalgae were added to our larvae containers. Microalgae were added at the same concentration as used in larvae rearing tanks and food levels were not limiting for the duration of the experiment.

On top of each tank, jars were attached to a wooden pole sitting horizontally across the tank. Each larvae jar was attached so that it was in a fixed position for the duration of the experiment, the position of the jar was noted so that the effect from placement in the tank could be ruled out. Jars were fully closed so that no water was shared between tanks and jars. The wooden pole was isolated from tank walls with polystyrene to avoid vibration propagation. One jar was located directly above the speaker and the other 4 jars were at the same distance from the center of the speaker (see Fig. 2).

After 24 h of exposure, larvae metamorphosis was checked using a dissecting microscope and the number of larvae that had cemented themselves to the substrates were counted. Metamorphosis was confirmed by gently blowing water over the larvae with a pipette to ensure that larvae were fixed to the substrate.

### Statistical analyses

A generalized linear mixed-effect model was created using the glmer function of the lme4 package^49^ in R version 4.1.3 (2022-03-10)^50^. As the response variable was binary (settled vs. not settled) we fitted a Bernoulli distribution using a logit link function. The predictor variables that were considered included sound treatment, date, speaker, tank, jar and jar position. First, we established a base model that included treatment and date as fixed effect variables and jars nested in the treatment-tank interaction as a random effect variable. We then included each of the other variables (i.e. speaker, tank and jar position) individually as fixed effects and compared model fit using the Akaike information criterion (AIC) and visually inspected model prediction plots. As the inclusion of any of those variables did not decrease the AIC value and had little to no effect on the effect sizes, we did not consider them in the final analysis. The assumptions of the model were met. See supplementary information for description of all model used (Table S5). Post hoc tests were performed using the emmeans function of the lsmeans package^51^ to calculate the marginal means adjusting *p*-values for multiple comparisons with Tukey's method and the pairs function was used to display pairwise comparisons.

## Results

### Playback

**Figure 3:**
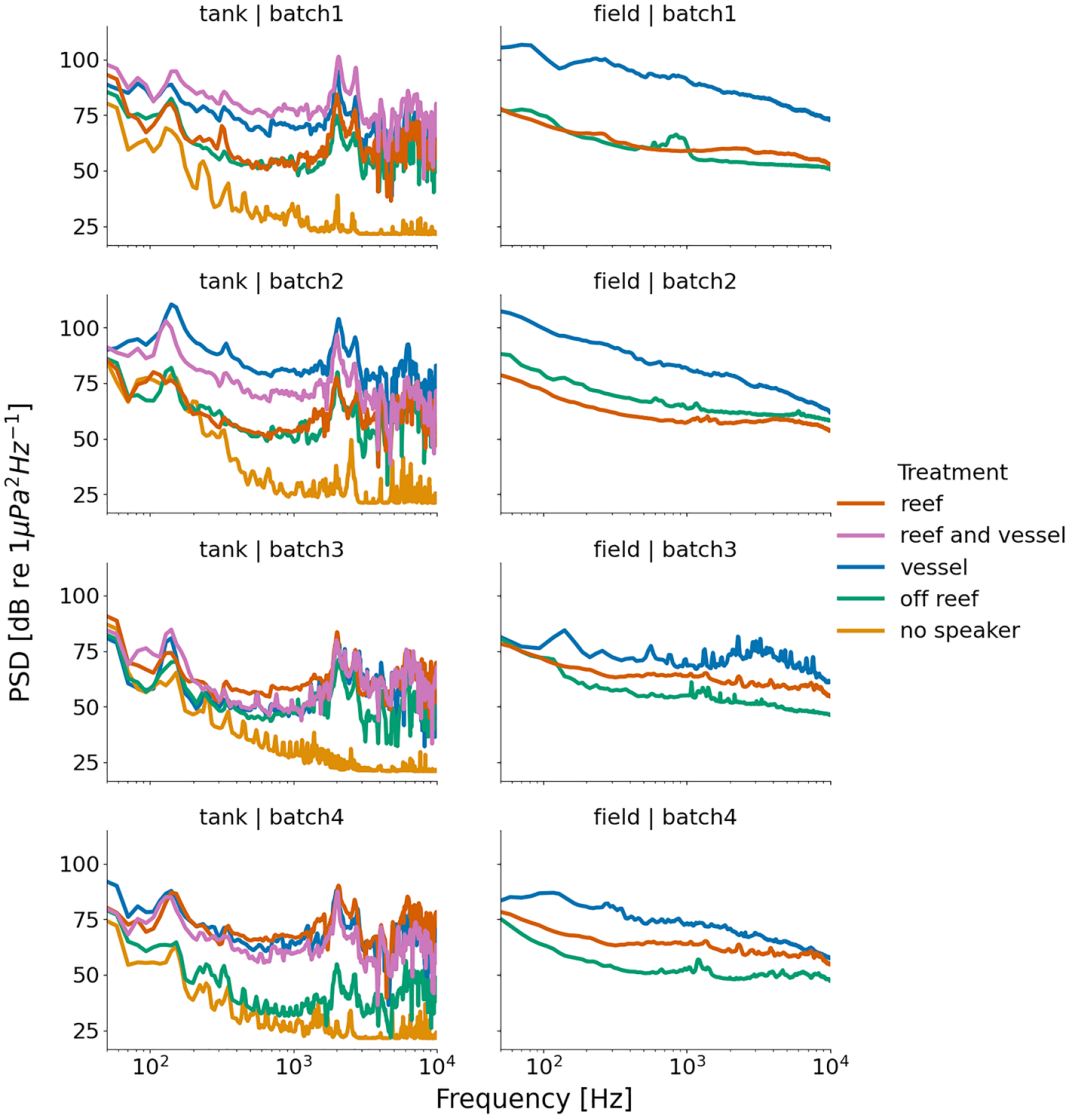
Comparison between the field and the tank recorded playback spectrum levels. No speaker refers to the tank recording when all the other playbacks were on (recorded in the tank with no speaker inside).

**Figure 4:**
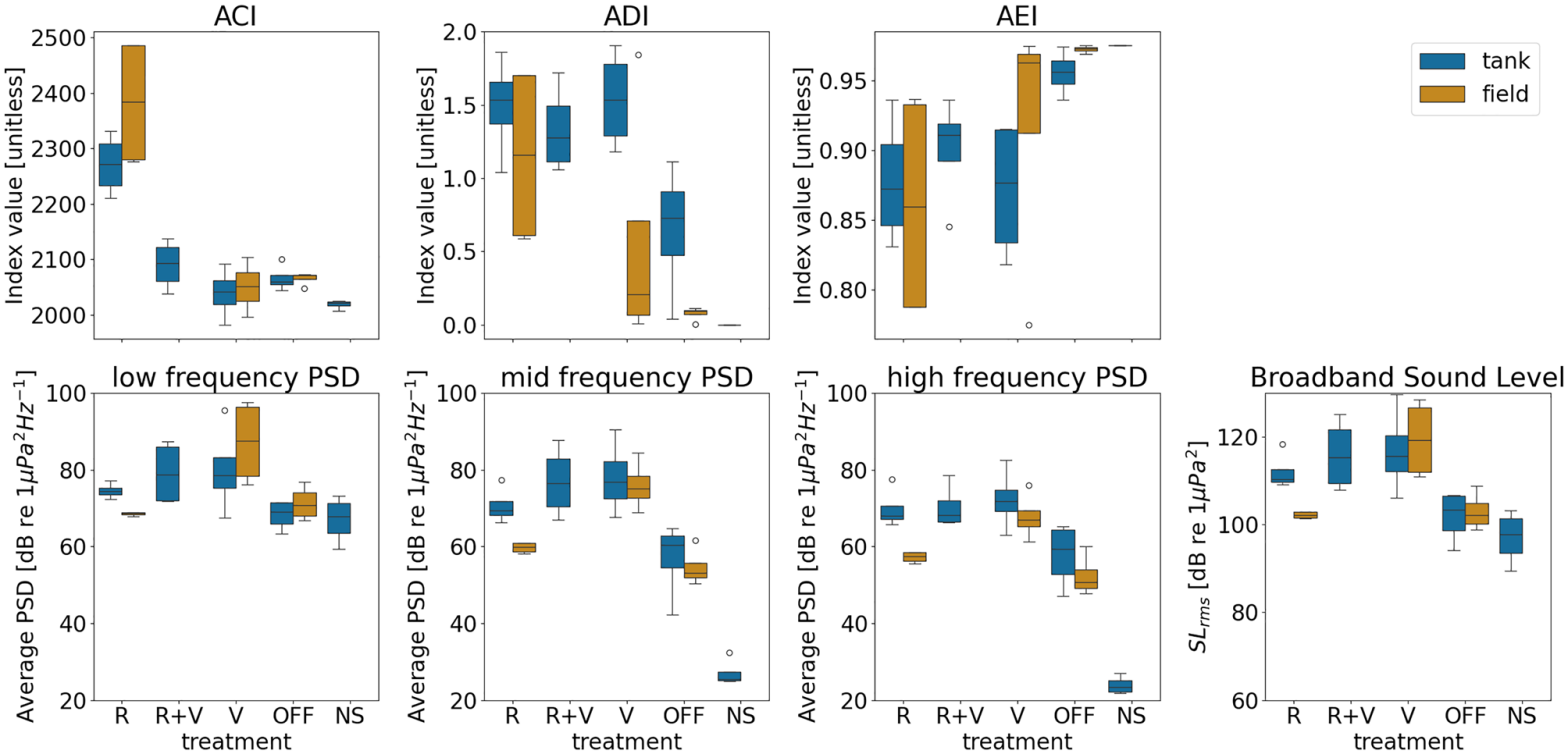
Comparison of the obtained acoustic metrics for the tank and the field recordings. The acoustic metrics include the acoustic complexity index (ACI), acoustic diversity index (ADI), and acoustic evenness index (AEI). NS = no speaker, OFF = off reef, R = Reef, R + V = reef and vessel, V = vessel. The number of data points for each treatment is 4. The definition and computation of each of the features are explained in Table S3.

The recorded sound in the tanks did not perfectly match the spectrum of the sounds recorded in the field due to the technical limitations of the reproduction equipment and the resonances that inevitably occur in tank-based experiments (Fig. 3). For example, sound levels were amplified at 2 and 7 kHz due to the speaker frequency response, with a dip at 5 kHz. A 50 Hz and its 3rd and 5th harmonic can be observed in the PSD of all the treatments, probably generated in the hydrophone due to electromagnetic interferences from the lab (not coming from the playback system and detectable by the larvae). Despite these limitations, when computing different acoustic metrics in tank and field sound recordings, similar trends were observed (Fig. 4). For example, ACI was higher for reef treatments compared to all other treatments in both tank and field sound recordings, and the PSD order from loudest to quietest for each batch was the same for field and tank recordings.

When analyzing the tank recordings, some faint cross-talk between tanks could be detected. This occurred only below 200 Hz, and it was most audible in the no speaker tank recording during the loudest moments of the neighboring tank. Nevertheless, the treatments remained very different and recognizable acoustically.

### Settlement rate

**Figure 5:**
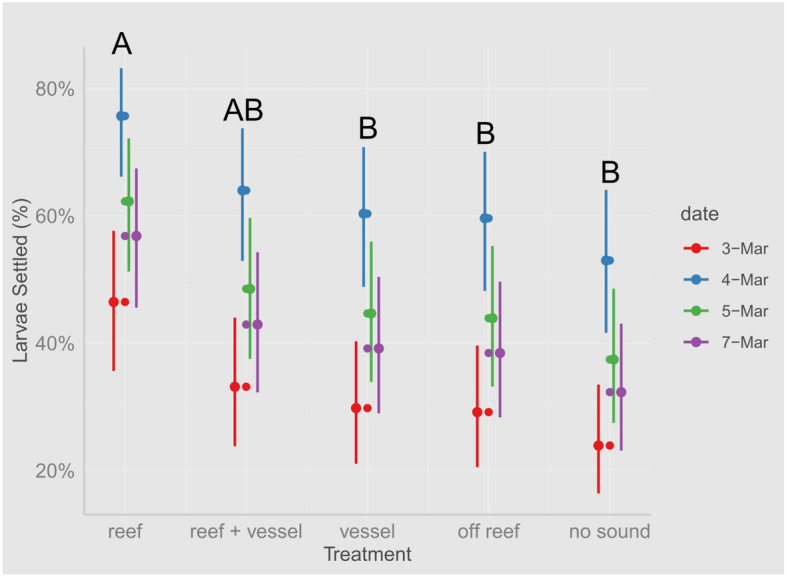
Prediction plots comparing predicted settlement across sound treatments and date of the experiment. Error bars represent 95% confidence intervals of the model prediction. Letters represent significant differences in the treatment, same letters mean no significant difference between treatments, but different letters indicate a significant difference.

Larvae settlement increased significantly in response to reef sound compared to vessel sounds (β = 0.715, SE = 0.260, *p* = 0.047), compared to off-reef sounds (β = 0.745, SE = 0.261, *p* = 0.034), and compared to the no speaker treatment (β = 1.015, SE = 0.262, *p* = 0.0010; Table 1). When vessel sound were added to the reef sound, the settlement rate decreased about 1.29 times compared to the pure reef sound (β = 0.560, SE = 0.259, *p* = 0.193), and was 1.09 times higher than in the vessel-only sound treatment (β = 0.155, SE = 0.261, *p* = 0.976). Comparisons among other treatments revealed only minor differences (Table 1). Vessels and off-reef sounds had very similar effects on settlement. The lowest settlement rates were observed in the no-speaker control treatment. Model predictions are plotted in Fig. 5.

**Table 1:** The results of the posthoc of the GLMER model 1 using all data and comparing all treatments.

Contrasting treatments	Estimate	SE	df	*z* ratio	*p* value
Reef—off reef	0.7455	0.261	Inf	2.86	**0.0344**
Reef—vessel	0.7154	0.260	Inf	2.750	**0.0471**
Reef—no speaker	1.0151	0.262	Inf	3.879	**0.0010**
Reef—(reef + vessel)	0.5600	0.259	Inf	2.164	0.1934
Off reef—vessel	0.0301	0.263	Inf	0.115	1
Off reef—no speaker	0.2695	0.263	Inf	1.025	0.8441
Off reef—(reef + vessel)	0.1855	0.261	Inf	-0.711	0.9541
No speaker—vessel	0.2997	0.264	Inf	-1.137	0.7869
No speaker—(reef + vessel)	0.4551	0.262	Inf	-1.738	0.4103
Vessel—(reef + vessel)	0.1554	0.261	Inf	-0.595	0.9758

Significant values (*p* ≤ 0.05) are in bold.

## Discussion

The results of our laboratory experiment showed increased settlement during exposure to sounds of conspecific oyster reefs for larvae of the oyster *Magallana gigas*, with settlement increasing 1.44 and 1.64 times under the oyster reef treatment compared to off-reef and no speaker control treatments, respectively. The settlement of larvae in response to vessel sounds, as well as the combined effects of reef and vessel treatments, did not show statistically significant differences compared to off-reef conditions and controls with no speaker.

### Preference for oyster reef sounds

Larvae of *M. gigas* consistently settle more readily when exposed to sounds of reefs inhabited by conspecifics. Our finding thus corroborates earlier research in fish, corals, and other oyster species, where larvae were found to increase settlement or orient more readily towards playback of reef sounds^8,36,52,53^. Yet the sound features that trigger this response still remain unidentified. In general, oyster and coral reefs exhibit higher sound levels and greater acoustic diversity than off-reef counterparts, due to increased soniferous biological activity including vocalizations of soniferous fishes and invertebrates, both passive or active, as well as the physical complexity of the reef^14,22^. It remains undecided in the literature if larvae can distinguish particular sounds from different habitats, or if there is simply a preference for certain acoustic features such as SPL^19^. The spectrum of reef sounds recorded for our study followed patterns similar to other oyster reefs^9,14^, and were louder than off-reefs in two out of four experiments. They also consistently presented a higher acoustic complexity, and higher evenness (lower AEI value) than off-reef areas. Compared to the vessel sounds, our reef sounds tended to have similar or lower PSD (depending on the vessel). Reef sounds were unique amongst the other treatments in their diversity, with consistently higher ACI and ADI values, and lower AEI values. This indicates that loudness (SPL) alone is not responsible for larval attraction, instead spectro-temporal patterns responsible for a high ACI may play a more important role. This conclusion can be corroborated in other marine invertebrates^17,25^. Pine et al.^17^ found that crab megalopae reduce metamorphosis (in comparison to natural habitat sounds) when exposed to wind turbine noise, but when the same turbine noises were played back at higher SPL, this did not result in any further changes to crab metamorphosis time. Leading to the conclusion that spectro-temporal characteristics were more relevant feature for megolopae attraction to habitat sounds than volume. Similarly, Gigot et al.^25^ found that scallop larvae reduced metamorphosis rates during drilling sounds, but increased metamorphosis rates when exposed to pile driving sounds. As both sounds were substantially louder than the no sound control, this further indicates the importance of temporal and spectral composition over simple preference for louder sounds. It would be incorrect to say that louder sounds are not a preferred sound feature for a number of invertebrates. Wilkens et al.^16^ found that when exposed to (the same) vessel sound at increasingly louder SPLs, mussel larvae increased settling at the louder treatments. Lillis et al.^19^ also conclude that louder reefs attract more coral settlers than quieter reefs. Based on the results of this present study and associated literature, a reasonable hypothesized could be made that both of these sound qualities (loudness and spectro-temporal patterns) are perceptible to *M.gigas* larvae, and the preferences for each may be highly species-specific and could be based on the preferred habit qualities. In comparison to *M.gigas* adults, who have previously been studied for their sense of hearing^35^, there is still not enough evidence to specify the range of acoustic frequencies detectable to larvae. Future research should therefore not only collect species specific data on acoustic feature detection, but also from different life stages.

### Vessel noises and larvae settlement

Our results show that exposure to vessel sounds alone did not manifest in any disruptions to settlement compared to off-reef or no speaker controls. This indicates that there may be no intrinsically negative reaction of *M. gigas* larvae to these vessel sounds. In marine invertebrates generally, vessel sounds induce a wide range of physiological and behavioral changes (see Solé et al.^18^ and Murchy et al.^54^ for reviews of vessels on marine invertebrates). Much of the evidence indicates a stress response to vessel noises, but cases where no reaction or a positive reaction to vessel sounds exist^18^. In the few cases where vessel noises are specifically tested on invertebrates during their settlement stage, reactions have varied. While coral larvae avoid vessel sounds^22^, mussels and sea squirt larvae increase settlement^24,55^.

While there was no significant difference between the reef treatment and the vessel + reef treatment, there appears to be a trend of reduced settlement in the vessel + reef treatment. The effect size is potentially ecologically relevant, with larvae being 1.29 times less likely to settle when vessel noise is added to the oyster reef sound. Treatments of reef + vessel noises did not differentiate significantly from off-reef and no speaker controls. Although this study does not provide conclusive evidence of habitat sounds being masked by vessel noises, it highlights the need for further investigation in this area. While cases of anthropogenic masking in other invertebrate settlement experiments remains unconfirmed, a recent study by McAfee et al.^7^ found that acoustic enriching experimental *Ostrea angasi* oyster reefs with reef sound was effective in low background noise areas, but ineffective in high background noise environments. However, the specific element of the background noise responsible for these results remains uncertain, as the term 'background noise' in the study encompassed all sounds within the soundscape (anthropogenic, geophysical, or biological). Looking past the biological response of overlaying vessels and reef sounds, acoustic characteristics of the reef sounds appeared to change with the addition of vessel noises in the recorded files. We observed a steep decrease in ACI, a mild decrease in ADI, and an increase in AEI, when vessel noises were added to reefs, although statistical confirmation is needed.

### Tank experiment limitations and future work

Lab-based sound exposure experiments are key as a first approach to test certain hypotheses, as they can be very controlled. External factors can be isolated, background ambient sound can be removed, and controls can be established from the same batch at the same time.

On the other hand, lab-based sound exposure experiments are incomplete in some aspects. For example, when presenting the whole picture of a soundscape reefs will change in their sonorific properties from a myriad of factors (time of day, time of year, etc.). Vessels, as well, are not likely to produce sound continuously at one location, as experienced in the playbacks. Nevertheless, to understand the acoustic basis for sound discrimination and use as a cue, it is necessary to use defined and identifiable sources so robust and direct conclusions can be extracted. In future work, once the contribution of acoustic characteristics are better understood, longer exposure experiments in the field should be done with more realistic soundscapes and environmental conditions.

Tank experiments also pose technical challenges in keeping playback sounds true to their original field recordings for several reasons. First, aquatic invertebrates, including oysters, sense particle motion rather than sound pressure^18^. Particle motion currently remains challenging to quantify, especially in small tanks. In the field, sound pressure and particle motion levels are strongly correlated, but that is not the case when close to the sound source and reflecting and pressure-relieving surfaces. Therefore, in smaller spaces such as a tank, the sound propagation will not necessarily be related to particle motion because the walls and surface will act as pressure release surfaces^56^. Hence, sound pressure measurements can be a poor indicator of the particle motion levels, especially close to the tank walls. However, the magnitude and direction of the particle motion are expected to differ substantially from the one the larvae would experience in the field.

Second, cross-talk between tanks is possible, as seen in the results Section. In this study, this cross-talk happened mostly at frequencies below 200 Hz, probably due to vibration propagation instead of air propagation, and it was mostly present during loud periods from the neighboring tank. However, tank recordings retained the acoustic characteristics necessary to make them distinguishable, as proven by the obtained results and by the manual analysis of the tank recordings.

Last, in tank sound experiments, the sound field can present great variations at small spatial scales. For this reason, the received sound levels were measured at all the jars when the speaker was playing white noise, giving very similar results, so the levels received at all the jars were considered the same treatment. We acknowledge that if the material would be available, doing simultaneously the tank recordings in all the jars would be of increased value, but still because the larvae were in jars of 100 ml, they still could be exposed to different sound fields (jars had a diameter of 4.4 cm and a length of 6.5 cm). Still, to account for these possible differences, jar position was included as a possible effect in the GML model.

To make causal inference, all parameters should be kept constant throughout and experiment. In the present study, we could not adhere to this principle in two aspects. Firstly, we used a different sound file for each of the treatments in each of the trials. This approach has the advantage that we make inferences about the effect of sound types, rather than specific sound files. Using multiple sounds offers a more realistic insight to each treatment, which enhances the ability to extrapolate, as explained in Section ‘Sound Treatments and Playback’. But it comes at the cost of limited power to detect a causal relationship as there is an additional confounding variation coming from the differences among sound files within sound types. Secondly, we had a limited number of speakers at our disposition. The trials were therefore conducted over several consecutive days and larvae could not be randomly assigned to the different trials and varied by age throughout the study. Our statistical analysis revealed that the date of the experiment had a significant effect on settlement. As the number of trials was limited, our design did not allow us to discriminate between the effect of experiment date and the effect of the variation among different sound files among treatment types. More and more extensive studies are needed to investigate which features of a specific sound type elicit the response in larvae settlement we observed in this study.

These limitations do not pose a problem for the current experiment, as our target was a proof-of-concept study into whether the settlement rate presented any differences when exposed to different acoustic stimuli in lab conditions, and to do so a fully controlled environment is necessary. Furthermore, the fluctuations over time at a large enough time scale represented in the ACI values are probably not affected by the dynamics of the tank resonances. Hence this cue remains valid in this experiment. While it necessary for future research to confirm our results under field conditions, proof of concept lab studies such as these, are essential first steps, as in the field, it is currently possible to control added sound, but not possible to remove other background sounds.

### Implications for reef restoration

Classic oyster reef mitigation and restoration projects focus on providing new hard substrates for wild larvae to settle, as well as supplying new adults to reefs, however, the importance of acoustic cues may be overlooked. Recently McAfee et al.^7^ used underwater speakers to enhance soundscapes on constructed reefs resulting in greater initial settlement of the oyster *Ostrea angasi*. If similar acoustic preferences are established for other species, these same techniques could be employed elsewhere. Our results indicate that *M. gigas* also responds to acoustic cues, and thus may respond positively to acoustic enrichment as a restoration strategy.

## Conclusion

We show that *M. gigas* larvae will settle more readily during playback of oyster reef sounds. The reef sounds were unique in being very acoustically diverse (high ACI), while other acoustic features, such as SPL varied among treatments. This suggests that oyster larvae may be able to detect complex spectro-temporal patterns in the soundscape rather than rely solely on SPL. Furthermore, we find that noise from vessels does not inhibit larvae settlement any more than the effect of off-reefs sounds or no speaker controls. We call for more research to replicate our findings in the laboratory in field experiments. More quantitative evidence is needed to determine if vessel noise (or other anthropogenic sounds) may affect oyster recruitment in ecologically realistic settings in the field.

### Supplementary Information

Supplementary Information.

## Data Availability

All data supporting the results in this paper will be archived upon publication.
