# Supplementary material for: Comparison of the effects of reef and anthropogenic soundscapes on oyster larvae settlement
Source: Sci Rep. 2024 May 31;14:12580. doi: 10.1038/s41598-024-63322-2 (PMC11143193; doi:10.1038/s41598-024-63322-2)
Supplement: Supplementary file 1 — Supplementary Information. [file 41598_2024_63322_MOESM1_ESM.docx]

# Supplementary Information

## Instrument Type

Table S1. description of all instruments used to collect acoustic data.

| Instrument type | Treatments | Model | Serial number | End to end sensitivity  [dB re. 1 μPa/V] | Sampling rate [kHz] |
| --- | --- | --- | --- | --- | --- |
| SoundTrap | REEF_34 | ST300 STD | 6045 | -176 | 24 |
| SoundTrap | REEF_1,  OFF_2 | ST300 STD | 6046 | -176.7 | 24 |
| SoundTrap | REEF_2 | ST300 STD | 6049 | -177.1 | 24 |
| SoundTrap | OFF_1 | ST300 STD | 6042 | -176.8 | 24 |
| RTSys | OFF_3, VESSELS_3 | RTSys RESEA + Colmar GP1190M-LP | EA-SDA14_2003003  GP1190M 134 | -176.98 | 96 |
| RTSys | VESSELS_1 | RTSys RESEA + Colmar GP1190M-LP | EA-SDA14_2003001  GP1190M 130 | -176.98 | 48 |
| RTSys | VESSELS_2 | RTSys RESEA + Colmar GP1516M-LP | EA-SDA14_2003003  GP1516M 185 | -164.98 | 48 |
| RTSys | VESSELS_4 | RTSys RESEA + Colmar GP1516M-LP | EA-SDA14_2003002  GP1516M 191 | -164.98 | 48 |

## Sound Treatments

Table S2. Different treatments with their corresponding acoustic data collection information. For each day of experiment (Day column in the Table), the reef + vessel combination was done by synthetically mixing the vessel and the reef recordings using Audacity.

| Treatment | Name | Location | Lat | Lon | Depth | Day | Description | Recordings Date times | Day moment |
| --- | --- | --- | --- | --- | --- | --- | --- | --- | --- |
| Reef | REEF_1 | Nieuweschild | 53.07 | 4.88 | X | 1 | Two times 10 minutes at different moments of twilight | 07/06/2021 02:40 | Civil twilight |
|  |  |  |  |  |  |  |  | 07/06/2021 05:20 | Day |
| Reef | REEF_2 | Kornwerderzand | 53.09 | 5.20 | 1.35 | 2 | 10 minutes | 30/05/2022 20:20 | Civil twilight |
| Reef | REEF_34 | Nieuweschild | 53.07 | 4.88 | X | 3,4 | 20 consecutive minutes | 07/06/2022 19:00 | Day |
| Off reef | OFF_1 | Nieuweschild | 53.07 | 4.88 | X | 1 | Sand area close to Nieuweschild reef. 20 consecutive minutes | 13/04/2021 20:39 | Astronomical twilight |
| Off reef | OFF_2 | Vlieland | 53.22 | 5.01 | 1.34 | 2 | Control recording for experiment of artificial reef. 10 minutes | 29/09/2022 06:19 | Day |
| Off reef | OFF_3 | Fairplay | 51.17 | 2.62 | 14.5 | 3 | 1 h of continuous off reef sound | 06/06/2021 04:19 | Day |
|  |  |  |  |  |  |  |  | 06/06/2021 04:42 | Day |
|  |  |  |  |  |  |  |  | 06/06/2021 04:54 | Day |
| Off reef | OFF_4 | Fairplay | 51.17 | 2.62 | 14.5 | 4 | 30 min continuous, with one distant vessel removed | 08/06/2021 15:46 | Day |
| Vessels | VESSELS_1 | Faulbaums | 51.33 | 2.51 | 15.2 | 1 | 3 different vessels: passing close by (11min), short trawling event (4min), loud and long anthropogenic sound (20min, trawling) | 08/06/2022 14:34 | Day |
|  |  |  |  |  |  |  |  | 08/06/2022 14:44 | Day |
|  |  |  |  |  |  |  |  | 06/06/2022 17:03 | Day |
| Vessels | VESSELS_2 | Grafton | 51.41 | 2.82 | 19.14 | 2 | Not repeated, 1h20min of continuous boat sounds. | 06/05/2022 14:39 | Day |
|  |  |  |  |  |  |  |  | 06/05/2022 15:31 | Day |
|  |  |  |  |  |  |  |  | 06/05/2022 15:51 | Day |
| Vessels | VESSELS_3 | Fairplay | 51.17 | 2.62 | 14.5 | 3 | 3 different vessels passing far (2, 5, and 5 min) | 06/06/2021 03:04 | Civil twilight |
|  |  |  |  |  |  |  |  | 06/06/2021 03:51 | Day |
|  |  |  |  |  |  |  |  | 06/06/2021 04:31 | Day |
| Vessels | VESSELS_4 | Buitenratel | 51.24 | 2.50 | 6.93 | 4 | Two different boats, one passing by and one close by anchored with constant sound. | 14/05/2022 18:55 | Day |
|  |  |  |  |  |  |  |  | 10/05/2022 17:13 | Day |

## Acoustic Features

Table S3. Computed acoustic features and their parameters. Broadband refers to all the frequencies from 0 to the Nyquist frequency (24 kHz). nfft stands for the number of Fast Fourier Transforms bins.

| Metric | Description | Frequency band [Hz] | Parameters | Reference |
| --- | --- | --- | --- | --- |
| SPL | Root mean squared value | broadband | NA | International Organization for Standardization, (2017)^1^ |
| PSD | Spectrum | broadband | nfft=4096 | International Organization for Standardization, (2017)^1^ |
| Low freq. | Average power spectral density | [0, 1000] | nfft=4096 | International Organization for Standardization, (2017)^1^ |
| Mid freq. | Average power spectral density | [1000, 5000] | nfft=4096 | International Organization for Standardization, (2017)^1^ |
| High freq. | Average power spectral density | [5000, 10000] | nfft=4096 | International Organization for Standardization, (2017)^1^ |
| ACI | Acoustic Complexity Index. Expresses the changes in amplitude in time within a frequency band.  Quantifies the acoustic irregularity and variability. | broadband | Hann window, nfft=4096, overlap=0.5 | Pieretti et al., (2011)^2^ |
| ADI | Acoustic Diversity Index.  Quantifies the evenness across frequency bands. A high value would be given if all the frequency bands have the same level, and a low value if one frequency band concentrates all the energy. | broadband | Hann window, nfft=4096, overlap=0.5 | Villanueva-Rivera et al., (2011)^3^ |
| AEI | Acoustic Evenness Index.  The opposite than ADI. Higher values indicate bigger unevenness in spectral distribution. | [0, 20000] | bin_step=500 dB_threshold=-50 | [Villanueva-Rivera et al., (2011)](https://www.zotero.org/google-docs/?broken=2nbUpH)^3^ |

## Speaker Assignment

The assignment of which speaker and which treatment would be used at each tank per batch was done randomly, resulting in the combinations listed in Table S4

Table S4. Distribution of treatment per tank and speaker. R+V stands for Reef+Vessel

| Day |  | Tank1 | Tank2 | Tank3 | Tank4 | Tank5 |
| --- | --- | --- | --- | --- | --- | --- |
| 1 | Treatment | NA | Vessel | Off reef | Reef | R+V |
|  | Speaker ID | NA | 3 | 2 | 1 | 4 |
| 2 | Treatment | Reef | NA | Vessel | R+V | Off reef |
|  | Speaker ID | 2 | NA | 4 | 1 | 3 |
| 3 | Treatment | Vessel | Off reef | R+V | NA | Reef |
|  | Speaker ID | 1 | 4 | 2 | NA | 3 |
| 4 | Treatment | R+V | Reef | NA | Off reef | Vessel |
|  | Speaker ID | 3 | 4 | NA | 1 | 2 |

## Playback Measurements

Prior to the experiment, we conducted recordings of white noise at all the jar positions to assess the differences in sound levels received at each jar. The jars were placed in a way that all of them except jar 3 were at the same distance and position from the speaker. The received PSD at each jar is very similar (see Figure S1). Nevertheless, we did not exclude these acoustic differences from having an impact on the settlement and for this reason jar position was investigated using a GLMER model, as described in the statistical analysis section.


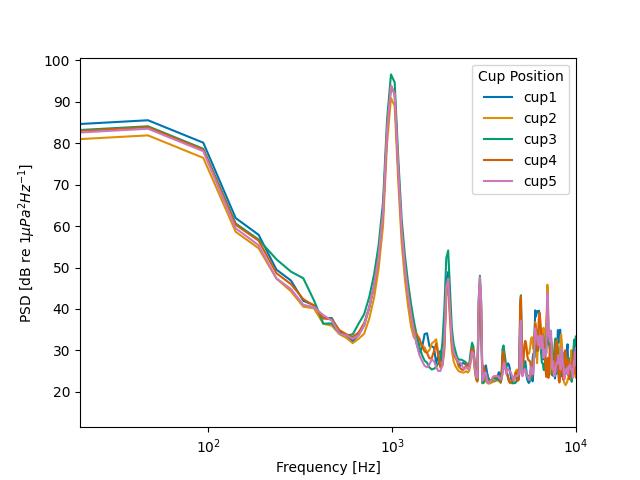


Figure S1. Power spectrum density received at all the jars when playing a white noise sound.

## Statistical Analysis

Raw data and statistical analysis are all available on the GitHub repository https://anonymous.4open.science/r/larvae-and-sound-A83F

Table S5. Description and output of statistical models used to determine if random variables had any effect to the base model.

|  | **Models compared** | **npar** | **ACI** | **BIC** | **logLik** | **deviance** | **Chisq** | **Df** | **Pr**  **(>Chisq)** |
| --- | --- | --- | --- | --- | --- | --- | --- | --- | --- |
| Assessing speaker effect on settlement | Base model | 10 | 960.6 | 1006.5 | -470.3 | 940.6 |  |  |  |
|  | Model including speaker | 13 | 964.25 | 1023.9 | -469.13 | 938.25 | 2.3471 | 3 | 0.5036 |
|  |  |  |  |  |  |  |  |  |  |
| Assessing tank effect on settlement | Base model | 10 | 1196.8 | 1244.9 | -588.41 | 1176.8 |  |  |  |
|  | Model including tank | 14 | 1203.9 | 1271.2 | -587.94 | 1175.9 | 0.9327 | 4 | 0.9198 |
|  |  |  |  |  |  |  |  |  |  |
| Assessing Jar position effect on settlement | Base model | 9 | 902.29 | 942.87 | -442.14 | 884.29 |  |  |  |
|  | Model including jar position | 13 | 906.11 | 964.73 | -440.06 | 880.11 | 4.1733 | 4 | .3831 |

## References

1. International Organization for Standardization. *ISO 18405:2017*. https://www.iso.org/cms/render/live/en/sites/isoorg/contents/data/standard/06/24/62406.html (2017).

2. Pieretti, N., Farina, A. & Morri, D. A new methodology to infer the singing activity of an avian community: The Acoustic Complexity Index (ACI). *Ecol. Indic.* **11**, 868–873 (2011).

3. Villanueva-Rivera, L. J., Pijanowski, B. C., Doucette, J. & Pekin, B. A primer of acoustic analysis for landscape ecologists. *Landsc. Ecol.* **26**, 1233–1246 (2011).
